# Normal Performance in Non-Visual Social Cognition Tasks in Women with Turner Syndrome

**DOI:** 10.3389/fendo.2018.00171

**Published:** 2018-05-04

**Authors:** David Anaki, Tal Zadikov-Mor, Vardit Gepstein, Ze’ev Hochberg

**Affiliations:** ^1^Department of Psychology, Bar-Ilan University, Ramat Gan, Israel; ^2^Gonda Multidisciplinary Brain Research Center, Bar-Ilan University, Ramat Gan, Israel; ^3^The Ruth Rappaport Children’s Hospital, Rambam Medical Center, Haifa, Israel; ^4^Rappaport Family Faculty of Medicine, Technion – Israel Institute of Technology, Haifa, Israel

**Keywords:** Turner syndrome, social cognition, visual-spatial skills, emotional expressions, theory of mind, faux-pas

## Abstract

Turner syndrome (TS) is a chromosomal disorder in women resulting from a partial or complete absence of the X chromosome. In addition to physical and hormonal dysfunctions, along with a unique neurocognitive profile, women with TS are reported to suffer from social functioning difficulties. Yet, it is unclear whether these difficulties stem from impairments in social cognition *per se* or from other deficits that characterize TS but are not specific to social cognition. Previous research that has probed social functioning in TS is equivocal regarding the source of these psychosocial problems since they have mainly used tasks that were dependent on visual-spatial skills, which are known to be compromised in TS. In the present study, we tested 26 women with TS and 26 matched participants on three social cognition tasks that did not require any visual-spatial capacities but rather relied on auditory-verbal skills. The results revealed that in all three tasks the TS participants did not differ from their control counterparts. The same TS cohort was found, in an earlier study, to be impaired, relative to controls, in other social cognition tasks that were dependent on visual-spatial skills. Taken together these findings suggest that the social problems, documented in TS, may be related to non-specific spatial-visual factors that affect their social cognition skills.

## Introduction

Turner syndrome (TS) is a genetic disorder, with an occurrence rate of approximately 25–50 per 100,000 females, resulting from a partial or complete absence of an X chromosome in a phenotypic female [a karyotype referred to as X-monosomy or 45, X; (1–5)]. This chromosomal absence leads to haplodeficiency of genes, which are normally expressed from both chromosomes. The physical appearance of women with TS is featured by short stature, webbed neck, and high-arched palate. They suffer from ovarian dysfunction, which leads to estrogen and androgen deficiency [e.g., Ref. (6)], and have significantly higher risks for hypertension, hypothyroidism, cardiac and renal defects, diabetes, and cancer. Treatment of TS includes induction of puberty by estrogen, and estrogen/progesterone replacement therapy in adulthood (1, 5).

Individuals with TS also demonstrate a unique psychosocial functioning profile. In childhood, girls with TS have difficulties in forming and maintaining social relations, and are more socially withdrawn than their typically developing (TD) peers. In adulthood, several studies also reveal that women with TS are less likely of achieving independent living and professional achievements that are on par with their education level [Ref. (7–10) but see Ref. (11)]. These problems may be the consequence of the social rejection that is experienced by individuals with TS, due to their syndrome-related physique and other abnormalities. However, they may be also related, at least in part, to their impairments in social cognition. Social cognition is an omnibus term which covers several psychological processes [Ref. (12, 13); see Ref. (14) for a detailed map of components of social cognition space]. Two important aspects of it are emotion perception and theory of mind: emotion perception is the ability to detect and perceive emotionally relevant information in one’s surroundings (15). Theory of mind is the ability to infer the contents of own and others mental states, including beliefs, intentions, emotions, thoughts, plans, and behavioral reactions (16). Both emotion perception and theory of mind are essential components of social functioning and, if impaired in TS, may account for their poor behavior in society.

Several studies have examined social cognition in TS. Specifically, women with TS were found to have difficulties, compared with normal controls, in recognition of emotions from facial expressions (17–20). Deficits were also detected in TS in recognizing emotional states from a restricted region of the face containing only the eyes (17). In addition, problems in theory of mind were also observed. For example, in one study participants were asked to describe short animations involving geometrical shapes (21–23). Description of these animations usually elicits mental-state descriptions, but TS women produced less mental-state descriptions than TD controls [see also Ref. (20) for similar results].

Although the accrued evidence indicates that women with TS are impaired in different aspects of social cognition it is noteworthy that all of the studies that examined the issue have used visual stimuli (e.g., faces, eyes, and animated shapes). This may be problematic since visual-spatial deficits have been widely recorded in women with TS [e.g., Ref. (24), see Ref. (3) for review]. Thus, it may be claimed that social cognition difficulties, demonstrated in TS, are restricted to visual stimuli and may be lacking, or at least attenuated when assessed through other modalities. The first goal of the present study was to investigate emotion perception and theory of mind in women with TS using tasks that consist of auditory-verbal stimuli. If social cognition impairments in TS are general, they would be seen across domains. However, if these difficulties arise or are exacerbated by the visual impairments in TS, comparable performance to TD controls is expected in non-visual social cognition tasks.

The second goal of the study was to examine the different aspects of theory of mind in TS. Theory of mind has been suggested to consist of two components, affective, and cognitive [e.g., Ref. (25)]. *Affective* theory of mind is the ability to acquire knowledge about the emotional states of others while *cognitive* theory of mind is the capacity of understanding other’s beliefs and thoughts. This distinction has been supported by several studies that have shown a dissociation between affective and cognitive theory of mind among different clinical populations [e.g., Ref. (26, 27)]. In women with TS, however, it was not explored, to the best of our knowledge, and it is yet to be determined how the affective and cognitive aspects of social cognition are expressed in TS compared with TD women.

## Materials and Methods

### Participants

Twenty-six women with TS and 26 TD controls participated in the study. The TS participants were recruited from the endocrinology clinic at the Ruth Rappaport Children’s Hospital, Rambam Medical Center. Twenty-four of them had chromosome Xp monosomy while two others had mosaic karyotypes. Fourteen women had taken growth hormone in childhood, and 20 received estrogen/progesterone replacement therapy in adulthood. The TS and TD groups were matched on age, education, and marital status (see Table 1). Both the TS and TD groups belong to the same sample as in the Anaki et al. (20) study. All participants had normal or corrected-to-normal vision. The study was conducted with approval of the hospital Institutional Review Board and after obtaining informed written consent from the participants, in accordance with the declaration of Helsinki.

**Table 1:** Demographic characteristics of the TS and typically developing groups.

	TS (*N* = 26)			TD (*N* = 26)			Significance
	Mean	SD	Range	Mean	SD	Range	
Age (years)	30.58	7.36	18–45	29.07	5.76	20–44	*t*(50) = 0.67, *p* > 0.51
Education (years)	13.90	1.90		14.04	1.71		*t*(50) = 0.25, *p* > 0.80
Marital status (% married)	35			38			χ^2^(1) = 0.08, *p* > 0.77
Performance IQ (WAIS-III Block Design SS)	8.88	2.41		11.38	2.86		*t*(50) = −3.4, *p* < 0.001
Verbal IQ (WAIS-III Similarities SS)	10.77	1.95		10.32	2.17		*t*(49) = 0.78, *p* > 0.37

*TS, Turner syndrome; TD, typically developing; WAIS-III, Wechsler adult intelligence scale III; SS, scaled scores*.

### Materials and Procedure

#### Verbal and Performance IQ

Verbal IQ and performance IQ were assessed with the Similarities and the Block Design subtests, respectively, taken from the Wechsler adult intelligence scale III (28). In the similarities subtest, participants are presented with pairs of words and are asked to identify the relationship between each pair. This subtest assesses verbal reasoning, concept formation capacities, and abstract thinking. The Block Design subtest consists of two-dimensional designs which the participants construct using three-dimensional blocks. This subtest reflects visual-motor analytic and synthetic skills.

#### Social Cognition Tasks

Three tasks were administered to address the two goals of the present study: first, an auditory expression task, in which TS and TD women were asked to identify different vocal expressions (29). In addition, participants also performed the *false belief task* (30), where short vignettes are presented and the participants have to infer the mental states of one of the characters. Finally, participants were given the *faux-pas* recognition task, where short stories are introduced and participants have to judge whether someone had said something which should not have been said (31). This task taps both affective and cognitive components of theory of mind as in order to understand that a wrong behavior has occurred one has to acknowledge two mental states: that of the addressee that feels insulted by the hurting utterance, and that of the addresser that does not know that he/she should not have said the faux-pas.

##### Auditory Expression Identification

The auditory stimuli used in this task were taken from the *Montreal affective voices* database, a standardized set of emotional vocal expressions designed for research on auditory affective processing with the avoidance of potential confound from linguistic content (29). From this set, we selected 6 expressions (happy, sad, fear, anger, surprise, and disgust), produced by 5 actors, and 5 actresses (60 vocalizations in total). The acoustic characteristics of the vocalizations were as follows: the median of the fundamental frequency (*f*0) was 333 Hz (SD = 103), the median sound duration was 1,094 ms (SD = 663 ms), and the median power was 72 decibels (SD = 8.56). The voices were presented on an IBM color monitor controlled by E-Prime software (Psychological Software Tools, Inc., 2000), implemented in an IBM PC-compatible computer.

Each trial began with a fixation point for 750 ms, followed by the auditory stimuli. The names of the six expressions appeared at the bottom of the screen, each with a corresponding number, and participants were asked to press the matching key. For each type of expression, the proportion of correct recognition was calculated.

##### The False Belief Task

The false belief task was comprised of two subtests; the first-order and the second-order false belief tasks. The former task assessed participant’s ability to infer that someone can have a mistaken belief that is different from the factual reality and the participant’s true belief. For example, Person A puts an object in a certain place in the presence of person B. Then, Person B leaves the room and Person A puts the object in a new location in the room. Person B then returns to the room. The participant is asked four questions: (a) a belief question—where Person B thinks the object is?, (b) a reality question—where the object is really located at the time of the return of Person B to the room?, (c) a memory question—what was the location of the object in the beginning?, and (d) a reference question—referring to some detail in the story which requires physical inference, in contrast to the mentalistic inference in the belief question.

The second-order false belief task evaluates one’s ability to understand what someone else thinks about what someone else thinks. For example, when Person B leaves the room he peeks back and sees how Person A moves the object, without the latter knowing that he is being seen. Person B then returns to the room. The participant is asked the same four questions as detailed above. Importantly, the belief question in this task probes the ability to grasp that individuals can represent the mental states of other people. Specifically, the question asked is: when Person B comes back to the room, where Person A will think person B thinks the object is? Participants completed four stories in each task (first- second-order false belief). Each story was read to the participants by the experimenter, followed immediately by the four questions.

##### Recognition of Faux-Pas

The faux-pas task consists of 10 stories in which a faux-pas situation occurs, and 10 control stories which depict interpersonal conflict but not of a faux-pas nature (30). After each story, the participant is asked two questions to ascertain whether he recognized a faux-pas situation (“Did someone say something he should not have said?” “Who said it?”). The two follow-up questions address whether the participant understands both the affective aspect of the faux-pas (i.e., “Why shouldn’t the individual in the story have said what he did?”), and the cognitive aspect of it, relating to the lack of intentionality from the viewpoint of the speaker (i.e., “Why do you think they said it?”). Finally, the participant is asked about an important detail in the story as a control condition to ensure that the story was understood. In the control stories, no faux-pas breach is made and the participants are required to provide a negative answer to the question about whether a faux-pas deed occurred. The control stories were not scored and were used as distracters. The stories were read to the participants but the printed version was placed before them to prevent any influence of memory, attention, or working memory. The answers, given by the participants, were written down by the experimenter.

### Research Design

The data were analyzed using independent samples *t*-tests for the verbal and performance IQ, as well as some demographic variables, with the participants’ group as a factor. The three social cognition tasks were analyzed using repeated-measures ANOVAs, with the participants’ group as a factor, and specific variables, unique for each task, as within-subject and dependent variables. The significance level was set to 0.05.

## Results

### Verbal and Performance IQ

Analysis of the similarities subtest did not reveal any differences between the TS and TD groups (see Table 1). In the Block Design subtest, however, TS women were less accurate than TD women.

### Auditory Expression Identification

**Figure 1:**
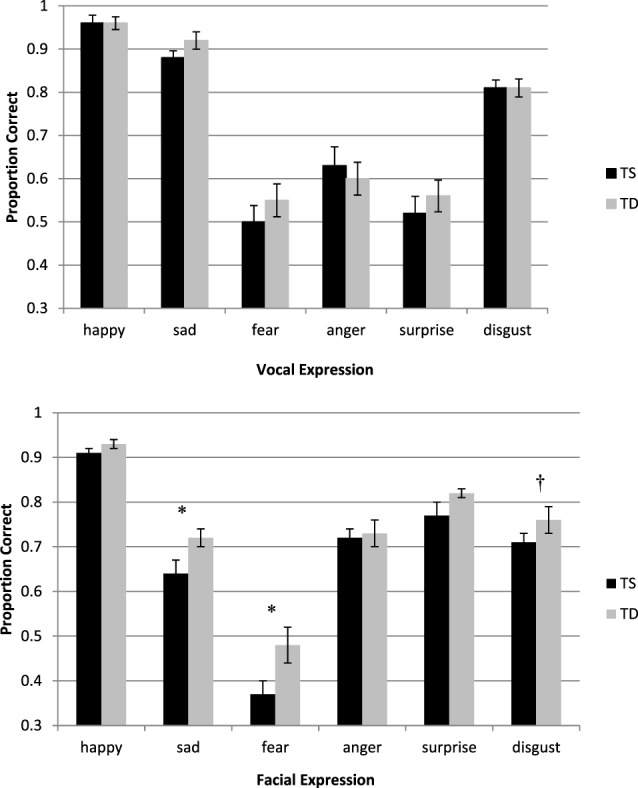
Top—accuracy in the auditory expression identification task as a function of group (TS and TD). Bottom—accuracy in the facial expression identification task as a function of group (TS and TD). The bottom graph is reprinted from Anaki et al. (20). Face perception in women with TS and its underlying factors, *Neuropsychologia, 90*, 274–285, with permission from Elsevier. Note—*p ≤ 0.05, ^†^significant only in a three-way interaction which included Group, Expression Type, and Morphing Level. TS, Turner syndrome; TD, typically developing.

Mean accuracy for the different conditions is presented in Figure 1. A repeated-measures ANOVA as a function of Group (TS and TD) and Expression Type (happy, sad, fear, anger, surprise, and disgust) was performed for the accuracy measures. The analysis revealed a significant main effect of Expression Type [*F*(5, 46) = 123.45, *p* < 0.0001, η^2^ = 0.93]. Pairwise comparisons (with Bonferroni corrections) revealed that the identification of happiness (M = 0.96, SD = 0.09), followed by sadness (M = 0.90, SD = 0.10), and disgust (M = 0.81, SD = 0.10) were the easiest to identify. The three other emotions, namely, surprise (M = 0.54, SD = 0.20), anger (M = 0.62, SD = 0.21), and fear (M = 0.53, SD = 0.20) were harder to identify and their accuracy level was comparable. Importantly, the group difference was not significant [*F*(1, 50) < 1], as well as the interaction between Group and Expression Type [*F*(4, 46) < 1].

### The False Belief Task

**Figure 2:**
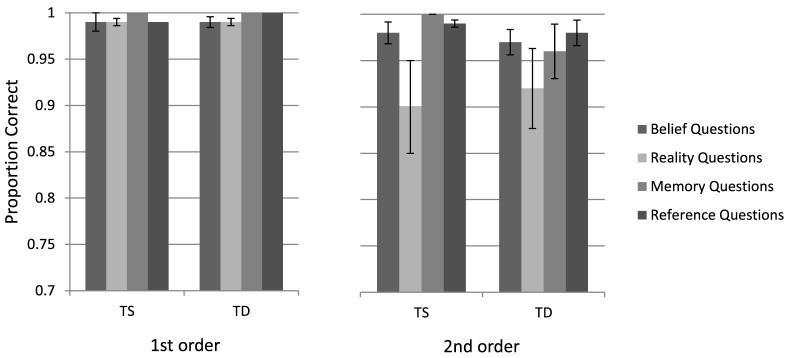
Turner syndrome (TS) and typically developing (TD) groups performance in the false belief task (first-order, second-order questions).

The mean accuracy for the different conditions is presented in Figure 2. A repeated-measures ANOVA on performance accuracy was performed as a function of Group (TS and TD), False Belief Task (first-, second-order), and Question Type (belief, reality, memory, and reference). The analysis yielded a main effect of the False Belief Task variable, resulting from higher accuracy for the first- (M = 0.99, SD = 0.07) than for the second-order questions [M = 0.96, SD = 0.09, *F*(1, 49) = 7.34, *p* < 0.01, η^2^ = 0.13]. Question Type was also found significant [*F*(3, 47) = 4.13, *p* < 0.01, η^2^ = 0.21], with reality questions less accurate (M = 0.95, SD = 0.09) than memory or reference questions (M = 0.99, SD = 0.04 and M = 0.99, SD = 0.03, respectively). Importantly, Group was not significant, neither by itself, as the main effect, nor by interaction with other variables (all *F*s < 1).

### Recognition of Faux-Pas

**Figure 3:**
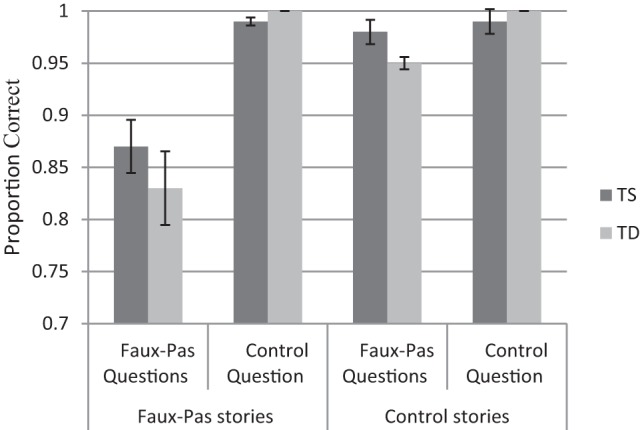
Turner syndrome (TS) and typically developing (TD) groups performance (proportions) in the recognition of faux-pas task.

A repeated-measures ANOVA on performance accuracy was performed as a function of Group (TS and TD), Story Type (faux-pas occurring/missing), and Question Type (faux-pas and control). The mean accuracy for the different conditions is presented in Figure 3.

Participants were more accurate in the control questions (M = 0.99, SD = 0.13) than in the faux-pas questions [M = 0.91, SD = 0.02, *F*(1, 50) = 63.81, *p* < 0.0001, η^2^ = 0.56]. In addition, participants were more accurate answering the questions in the non-faux-pas conditions (M = 0.98, SD = 0.05) than in the faux-pas conditions [M = 0.93, SD = 0.14, *F*(1, 50) = 17.52, *p* < 0.0001, η^2^ = 0.26]. Finally, a Story Type X Question Type interaction was observed [*F*(1, 50) = 19.18, *p* < 0.0001, η^2^ = 0.28], indicating that for control questions accuracy was comparable across story type [M = 0.97, SD = 0.06 and M = 0.99, SD = 0.01, for faux-pas and non faux-pas stories, respectively, *t*(51) = 0.57, *p* < 0.0001]. However, accuracy for the faux-pas questions was lower in scenarios where faux-pas occurred compared with when it did not occur [M = 0.85, SD = 0.16 and M = 0.99, SD = 0.01, respectively, *t*(51) = 4.35, *p* < 0.0001]. Importantly, as in previous tasks, the Group variable was not significant by itself or with other variables.

**Figure 4:**
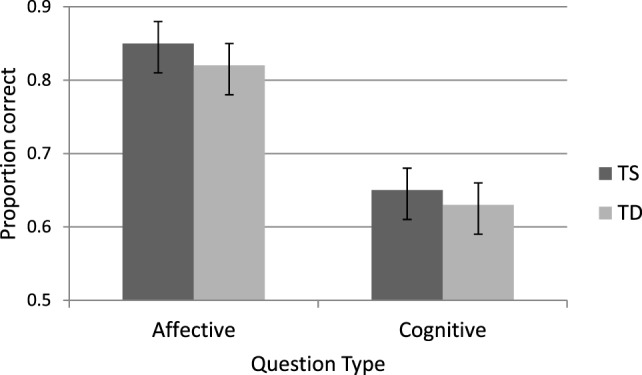
Accuracy in faux-pas affective and cognitive questions for Turner syndrome (TS) and typically developing (TD) women.

A second ANOVA analysis was performed on the two faux-pas questions that refer to the affective and cognitive aspects of the faux-pas. As can be seen in Figure 4 responses to the affective questions were more accurate than the cognitive ones [M = 0.83, SD = 0.17 and M = 0.64, SD = 0.21, respectively, *F*(1, 50) = 126.75, *p* < 0.0001, η^2^ = 0.72]. TS women were as accurate as TD women for both affective and cognitive questions (*F* < 1).

## Discussion

The present study aimed to examine social cognition in TS. Although this topic was addressed to some extent in TS research, past studies have used tasks that required reliance on the visual modality (e.g., the perception of facial expressions). However, in the current study, the social cognition tasks entailed auditory-verbal capacities. In addition, the present study sought to explore affective and cognitive aspects of theory of mind and whether a unique pattern may characterize TS. The findings revealed a comparable performance of TS and TD women in all the three tasks examined. Specifically, the performance of women with TS was similar to TD women in recognizing auditory expressions, in identifying situations in which a faux-pas behavior occurred and in mentalizing the thoughts of different individuals described in short vignettes. In addition, both groups showed more cognitive than affective errors in the faux-pas task, yet no difference was found between TS and TD women in understanding cognitive and affective aspects of the theory of mind. We believe that these findings shed new light on the social cognitive capacities of women with TS, and provide an alternative interpretation of the difficulties that they experience in social functioning. In the following, we will elaborate on the theoretical implications of the present results.

Abundant findings suggest that social functioning is compromised in TS, at least to some extent [e.g., Ref. (32)]. Girls with TS are involved in fewer social activities than their peers and exhibit lower than normal competence in social interactions and interpersonal relationships (33–35). They are evaluated by their caregivers as performing poorly in social awareness, cognition, and communication (7). Women with TS are reported to have fewer intimate partners, they stay longer at their parental homes and marry at a lower rate than their control peers [e.g., Ref. (36–38)]. Some studies find that women with TS perceive themselves as having less social competence compared with controls, and report higher levels of shyness and social anxiety (39, 40).

Several accounts were proposed to explain the social difficulties that exist in TS. One hypothesis attributes these problems to social stigma arousing from body deformities related to the syndrome, such as short stature, webbed neck, and other physical deformations (e.g., cubitus valgus). However, studies have failed to find a relationship between physical appearance and social performance (41). Another potential underlying factor of the deficient social functioning may be related to the poor psychological well-being of individuals with TS due to the burden of coping with the medical, cognitive, and physiological consequences of the syndrome (42). Finally, the impaired social functioning in TS can be part and parcel of the disorder itself, reflecting an inherent deficit in social cognition [e.g., Ref. (3, 7, 32, 43)].

Past studies have provided evidence favoring this last account. For example, women with TS are less accurate than normal controls in recognizing facial expressions, perceiving eye gazes, and inferring mental states from animated objects (17–21, 44). However, the social cognition impairment hypothesis is based mainly on tasks that required visual-spatial capacities, known to be impaired in TS. Thus, these latter findings supporting specific social cognition impairment in TS may be confounded with non-specific variables such as visuospatial factors. This possibility raises an alternative account that social functioning may be hindered by non-specific factors that are prevalent in TS, such as visuospatial difficulties (5). In order to disentangle the two accounts, the present study sought to assess social cognition in TS with non-visual stimuli. We conjectured that if TS women will show difficulties in these tasks as well, it will support the notion that social cognition is impaired in TS, regardless of modality or specific TS-related deficits. On the other hand, equivalent performance in these tasks of TS women and TD controls will support the claim that social cognition deficits in TS stem, at least to some degree, from other impairments that characterize TS.

The present findings are not compatible with the account of social cognition deficits in TS. In addition, the findings are at odds with our previous study, where the same cohort of TS women was found to be impaired, compared with TD women, in social cognition tasks (20). Specifically, in the facial expression recognition task, they were less accurate than control participants in identifying facial expressions, especially fearful, sad and, in some conditions, disgust expressions (see Figure 1). In addition, in the animated triangles task, women with TS were less accurate in providing descriptions of the animations. Moreover, their responses contained less mental-states portrayals of the unfolding events and more external physical descriptions of the objects’ movements.

A couple of potential interpretations can be offered to explain this discrepancy. First, the tasks may have differed in difficulty and therefore TS impairments emerged only in the more challenging tasks, namely, the facial expression and animated triangles task. However, this interpretation does not seem plausible since some of the tasks were similar in difficulty, and still yielded different results. For example, the facial visual expression and auditory expression identification tasks were of similar difficulty, as indicated by the same level of accuracy obtained by TD women. TS women performed the auditory expression identification task as well as their controls. Yet, they displayed reduced proficiency in the visual expression identification task, compared with their own performance in their auditory task. The performance of the two groups in the faux-pas task was also far from reaching ceiling effects. Thus, the enhanced performance of TS women in the non-visual social cognition tasks compared with the visual tasks does not appear to result from differential levels of tasks’ difficulty.

An alternative account to the discrepancy observed between the different tasks attributes the conflicting findings, regarding TS social cognition capacities, to the different modalities that have been used. According to this interpretation, TS impairments in social cognition tasks are exacerbated in tasks that are based on the visual modality. This is because TS individuals are plagued by visual-spatial impairments, reflected empirically in their lower Wechsler Performance IQ score compared with their normal Wechsler Verbal IQ score [see Ref. (3) for review, see Ref. (45)]. In contrast, their social cognition deficits are less discernible (or even non-existent) in tasks based on non-visual stimuli. Indeed, in our TS cohort we have found poorer TS performance in the Block Design subtest of the Wechsler, which is part of the Performance IQ score. The TS women were also less accurate than the TD women in performing subtests from the Birmingham Object Recognition Battery that assesses different levels of visual object perception (46). However, both TS and TD women were comparable in their performance in the Wechsler’s Similarities subtest. Thus, our comprehensive findings appear to support the view that social cognition may not be impaired *per se* in TS but rather affected by non-specific visual impairments that characterize this syndrome.

**Figure 5:**
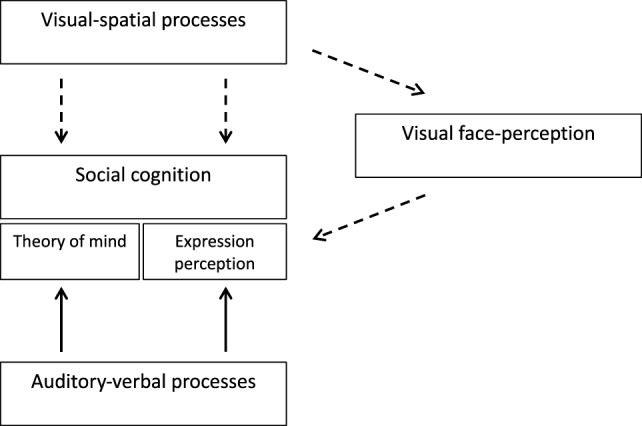
A theoretical model of social cognition in Turner syndrome (TS) and its relationship to other perceptual and cognitive capacities. The broken lines represent TS impairments while the full lines represent intact capacities.

The proposed explanation suggests that social cognition and its various facets (e.g., theory of mind, expression perception) are independent abilities (14). However, social cognition functioning harness other processes as well, processes that are involved in social cognition but are not specific to it. The auxiliary processes that have been the focus of the present study and Anaki et al. (20) study are visual-spatial, auditory-verbal, and facial perception capacities (Figure 5). In TS auditory-verbal capacities are relatively intact and therefore they are able to support aural and linguistic aspects of social cognition. In contrast, visual-spatial processes are impaired in TS, and consequently, the direct or indirect support (namely through face perception), that they can provide to social cognition, is more limited. Due to the substantial involvement of visuospatial abilities in social cognition, the social behavior of women with TS will be compromised. Indeed, partial compensation would be possible through other sensory modalities but it would not allow the full practical expression of their social skills.

The claim that social cognition by and of itself is not impaired in TS is purportedly inconsistent with neuroimaging studies conducted in TS, that have shown abnormalities in several brain areas, some of them known to be involved in emotional processing. Specifically, structural imaging studies in TS have found greater brain volumes in the amygdala, cingulate, and insular cortices, but also reduced cortical thickness in other brain areas, such as left frontal lobe and bilateral parahippocampal gyrus [Ref. (1, 7, 24, 43, 47, 48), see Ref. (49) for review]. In a recent study, Lepage et al. (50) found that socio-emotional functioning in TS [measured with the Emotional Quotient Inventory (51)] was related to their aberrant brain morphological structures of the social brain. In addition, functional imaging studies have found anomalous patterns of amygdalar activation in response to fearful stimuli (52), as well as reduced neural activity in frontal areas, such as the anterior dorsal anterior cingulate cortex and the dorsolateral prefrontal cortex (44). These findings appear to be more consistent with the claim that the social problems in TS are due to anomalies in brain regions related to social processing.

However, as already pointed out [e.g., Ref. (50)], it is hard to determine whether the brain’s unique morphological and activation patterns, observed in TS, are the cause of their social difficulties or the consequence of it. Moreover, the inclusion of other factors claimed to be involved in social cognition, such as visual-spatial skills, establishes a complex network which consists of potential mediating and interactive relationships among different variables. For example, TS visual-spatial deficits, apparent at birth, may influence the structural and functional development of social-related regions, and consequently social behavior. Alternatively, an atypical TS social brain could exist from an early stage, but visual-spatial difficulties could shape its trajectory and accelerate the rate of its abnormal development. For now, the present results emphasize the importance of visual-spatial factors in the development of TS deficits in social cognition. Future longitudinal studies are therefore required to determine the exact structure and functioning of the social brain in girls and women with TS and its impact on their social functioning.

## Ethics Statement

This study was carried out in accordance with the recommendations of the Ruth Rappaport Children’s Hospital Institutional Review Board with written informed consent from all subjects. All subjects gave written informed consent in accordance with the Declaration of Helsinki. The protocol was approved by the Ruth Rappaport Children’s Hospital Institutional Review Board.

## Author Contributions

All authors contributed to the conception and design of the study; VG and ZH selected the TS sample; TZ-M conducted the research and collected the data; TZ-M and DA performed the statistical analysis; DA wrote the first draft of the manuscript. All authors contributed to the manuscript first draft, read, and approved the submitted version.

## Conflict of Interest Statement

The authors declare that the research was conducted in the absence of any commercial or financial relationships that could be construed as a potential conflict of interest.
